# Гипофизит во время беременности с исходом в стойкий несахарный диабет

**DOI:** 10.14341/probl13384

**Published:** 2024-09-15

**Authors:** Л. К. Дзеранова, Е. А. Пигарова, С. Ю. Воротникова, А. А. Вознесенская

**Affiliations:** Национальный медицинский исследовательский центр эндокринологии; Национальный медицинский исследовательский центр эндокринологии; Национальный медицинский исследовательский центр эндокринологии; Национальный медицинский исследовательский центр эндокринологии

**Keywords:** центральный несахарный диабет, беременность, лимфоцитарный гипофизит

## Abstract

Аутоиммунный/лимфоцитарный гипофизит является одной из редких причин развития центрального несахарного диабета у взрослых пациентов и наиболее часто встречается среди женщин во втором или третьем триместрах беременности.

В многочисленных исследованиях показано, что лимфоцитарный гипофизит характеризуется весьма вариабельной клинической картиной с развитием неврологической симптоматики, нарушений со стороны зрения и гипопитуитаризма с частичным или полным выпадением функций гипофиза, а также рядом особенностей при магнитно-резонансной томографии (МРТ).

Изолированный лимфоцитарный инфудибулонейрогипофизит встречается значительно реже и затрагивает заднюю долю и ножку гипофиза с клинической картиной несахарного диабета.

В приведенном клиническом случае описывается развитие гипофизита у беременной пациентки с преимущественным поражением задней доли гипофиза и исходом в несахарный диабет, сохраняющимся через 6 лет после беременности и родов.

В статье рассмотрены аспекты дифференциальной диагностики несахарного диабета у беременных, а также особенности инструментальной диагностики и подходов к лечению гипофизита.

## Abstract (en)

Autoimmune/lymphocytic hypophysitis is one of the rare causes of central diabetes insipidus in adults and is most common among women in the second or third trimester of pregnancy. Numerous studies have shown that lymphocytic hypophysitis is characterized by a very variable clinical signs with the development of neurological symptoms, visual disturbances and hypopituitarism with partial or complete loss of pituitary function, as well as a number of features in magnetic resonance imaging (MRI). Isolated lymphocytic indibuloneurohypophysitis occurs in fewer cases and involves the posterior lobe and stalk of the pituitary gland with a clinical presentation of diabetes insipidus. The above clinical case describes the development of hypophysitis in a pregnant woman with a predominant lesion of the posterior pituitary gland and an outcome in diabetes insipidus, which persists 6 years after pregnancy and childbirth. In the article some aspects of the differential diagnosis of diabetes insipidus in pregnant women, as well as instrumental diagnosis and treatment approaches of hypophysitis are discussed.

## АКТУАЛЬНОСТЬ

Несахарный диабет (НД) во время беременности представляет собой относительно редкое состояние с распространенностью примерно 2–6 случаев на 100 000 беременных пациенток [1].

Известно, что несахарный диабет у беременных может развиваться в результате повышения активности фермента вазопрессиназы, секретируемой плацентой, и в таком случае носит название гестационного несахарного диабета, и несколько реже может быть следствием различных патологических процессов в головном мозге (центральный несахарный диабет, ЦНД) или резистентности почек к действию вазопрессина (нефрогенный несахарный диабет) [2].

Отдельный интерес представляют случаи развития центрального несахарного диабета у беременных на фоне аутоиммунного (лимфоцитарного) гипофизита, как правило, характеризующегося частичным или полным выпадением функций адено- и/или нейрогипофиза, а также рядом неврологических и офтальмологических нарушений [3].

В приведенном клиническом случае описывается развитие гипофизита у беременной пациентки с преимущественным поражением задней доли гипофиза и исходом в несахарный диабет, сохраняющимся через 6 лет после беременности и родов. Данный клинический пример интересен тем, что в большинстве описанных ранее случаев гипофизита у беременных отмечалось стойкое нарушение функции аденогипофиза или обеих долей гипофиза [4–8], в то время как изолированное поражение задней доли с развитием инфундибулонейрогипофизита и, как следствие, несахарного диабета, встречалось значительно реже [9][10]. Данное обстоятельство требует большей осведомленности о возможных клинических, лабораторных и инструментальных признаках гипофизита и подходах к его лечению среди эндокринологов, акушеров-гинекологов.

## ОПИСАНИЕ СЛУЧАЯ

Пациентка У., 26 лет, впервые обратилась в ГНЦ РФ ФГБУ «НМИЦ эндокринологии» Минздрава России в 2017 г. на сроке 20–21 неделя беременности с жалобами на выраженную жажду с потреблением до 6,0–6,5 л жидкости в сутки, учащенное обильное мочеиспускание с выделением до 6,0 л жидкости в сутки, никтурию.

Из анамнеза известно, что с 14-й недели беременности пациентку стали беспокоить выраженные головные боли по типу «обруча» с ощущением «пульсации в глазнице справа», по рекомендации невролога получала терапию триптанами, на фоне чего болевой синдром кратковременно купировался. Через некоторое время пациентка отметила появление отека век справа, при осмотре офтальмологом выявлено снижение остроты зрения на правый глаз, сужение полей зрения биназально.

В рамках обследования по поводу головных болей и нарушения полей зрения на 17–18 неделе беременности пациентке выполнена МРТ головного мозга без контрастного усиления, по результатам которой выявлены признаки аденомы гипофиза размерами 10x15x20 мм с супраселлярным ростом и умеренной компрессией хиазмы, нейрогипофиз не дифференцировался (рис. 1, 2).

**Figure:**
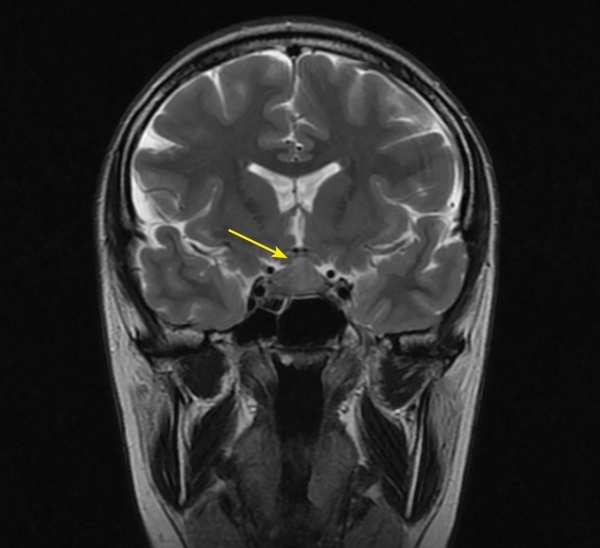
Рисунок 1. МРТ головы без контрастного усиления, Т2-взвешенное изображение, коронарная (фронтальная) проекция. МР-картина «аденомы» гипофиза размерами 10x15x20 мм с супраселлярным ростом и умеренной компрессией хиазмы (изменения указаны стрелкой).

**Figure:**
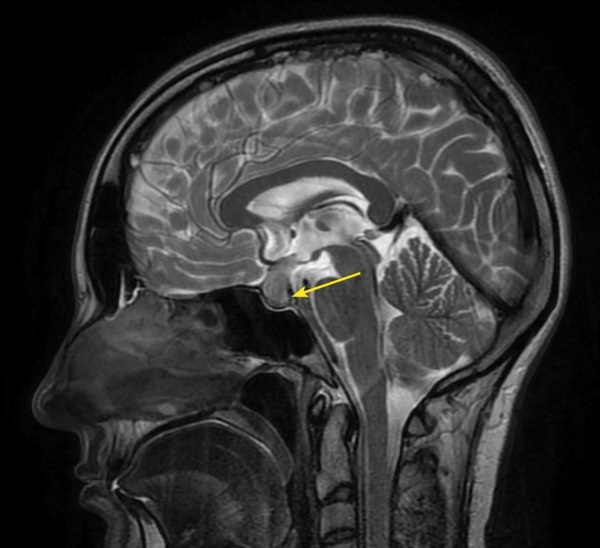
Рисунок 2. МРТ головы без контрастного усиления, Т2-взвешенное изображение, сагиттальная проекция. Нейрогипофиз не дифференцируется (изменения указаны стрелкой).

В связи с сохраняющимися головными болями и отеком век справа пациентка самостоятельно инициировала терапию дексаметазоном внутримышечно по 4–8 мг 1 раз в 3 дня с 19–20 недели беременности. Лечение дексаметазоном сопровождалось уменьшением отека век, улучшением зрения, купированием головных болей. При повторном осмотре офтальмологом через несколько недель офтальмологических нарушений не выявлено.

На сроке 18–19 недель беременности пациентка впервые отметила повышенную жажду и частое обильное мочеиспускание. Проводилось исключение сахарного диабета (СД), при обследовании пациентки на 18-й неделе беременности уровень гликемии натощак составил менее 5,1 ммоль/л. В общем анализе мочи выявлена низкая относительная плотность (1000 г/мл). В это же время по результатам гормонального исследования нельзя было исключить развитие вторичного гипотиреоза и вторичного гипокортицизма: ТТГ — 0,022 мЕд/л (0,4–4,0), Т4 свободный (свТ4) — 9,12 пмоль/л (9,0–19,0), кортизол утром — 39 нмоль/л (101–535), по результатам анализов пациентка к врачу не обращалась.

При первом обращении в ГНЦ РФ ФГБУ «НМИЦ эндокринологии» Минздрава России (20–21 неделя беременности) повторно проведенное гормональное исследование не выявило признаков вторичного гипотиреоза и гипокортицизма: ТТГ — 1,89 мЕд/л (0,4–4,0), свТ4 — 12,9 пмоль/л (11,5–22,7), АКТГ утром — 14,2 пг/мл (0,0–46,0), кортизол утром – 564 нмоль/л (101–535), при этом относительная плотность мочи оставалась низкой — 1000 г/мл. По результатам перорального глюкозотолерантного теста, проведенного на 24 неделе беременности, данных за нарушение углеводного обмена получено не было.

На фоне продолжающейся терапии дексаметазоном, по данным МРТ головного мозга, отмечался регресс изменений хиазмально-селлярной области с утолщением воронки гипофиза, отсутствием сигнала от нейрогипофиза (рис. 3, 4).

**Figure:**
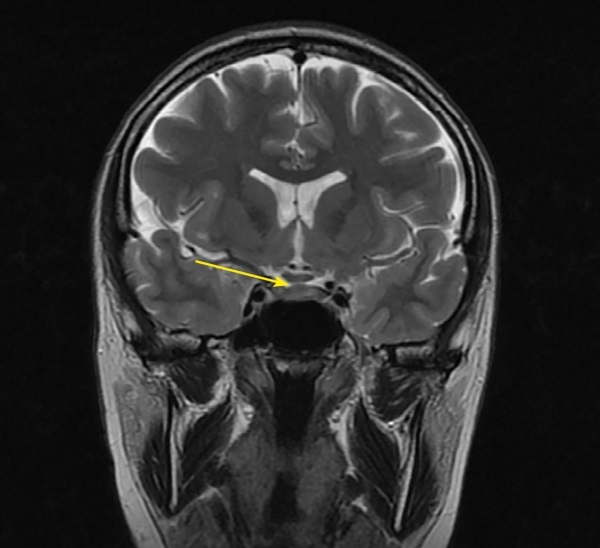
Рисунок 3. МРТ головы без контрастного усиления, Т2-взвешенное изображение, коронарная (фронтальная) проекция. Сохраняется утолщение ножки гипофиза (изменения указаны стрелкой).

**Figure:**
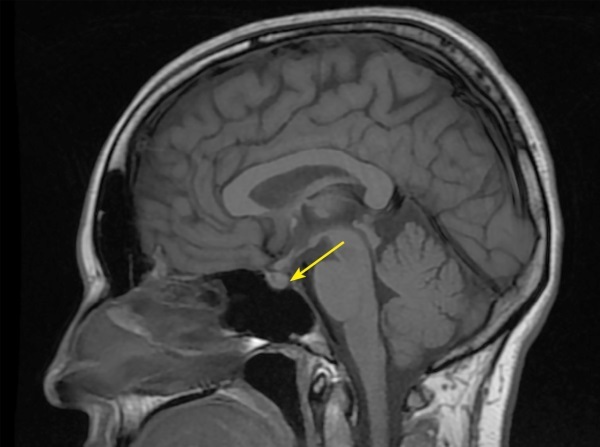
Рисунок 4. МРТ головы без контрастного усиления, Т1-взвешенное изображение, сагиттальная проекция. Нейрогипофиз не дифференцируется (изменения указаны стрелкой).

С учетом характерной МР-картины, анамнеза и сохраняющихся жалоб пациентки на повышенную жажду и обильное, частое мочеиспускание состояние расценено как гипофизит с развитием несахарного диабета. Рекомендован прием подъязычных таблеток десмопрессина по 30 мкг 2 раза в сутки, на фоне чего пациентка отметила значительное улучшение самочувствия с нормализацией объема потребляемой жидкости и режима мочеиспускания. В дальнейшем самостоятельно уменьшила дозу десмопрессина до 15 мкг 2 раза в сутки.

На 35–36 неделе начались преждевременные роды, с учетом рекомендаций нейрохирурга родоразрешение проведено путем кесарева сечения, родился мальчик, 7/7 баллов по шкале Апгар, весом 2120 г, длиной тела — 42 см. При первом осмотре отмечены признаки недоношенности, показатели дыхания и сердцебиения — в пределах нормы. В раннем неонатальном периоде отмечалось развитие гипогликемии у ребенка (клинически — вялость движений, слабый сосательный рефлекс), вероятнее всего, вследствие самостоятельно продолженной пациенткой терапии дексаметазоном (внутримышечные инъекции) вплоть до родоразрешения, а также недоношенной беременности. Проводилось внутривенное введение 5-процентного раствора глюкозы. Лактация прервана вскоре после родоразрешения по инициативе пациентки агонистами дофамина, регулярный менструальный цикл восстановился через месяц после родов и сохраняется в настоящее время.

Пациентка повторно обратилась в ГНЦ РФ ФГБУ «НМИЦ эндокринологии» через 6 лет после родов в июне 2023 г. на фоне терапии десмопрессином 15 мкг 2 раза в сутки для подтверждения наличия НД, исключения гипопитуитаризма и коррекции терапии.

В ходе обследования на фоне пропуска приема вечерней дозы десмопрессина и ограничения приема жидкости с 21:00 ч. в анализах крови утром отмечалась гипернатриемия (натрий крови — 148,4 ммоль/л (136,0–145,0 ммоль/л), повышенная осмоляльность плазмы — 302 мОсмоль/кг (280–300 мОсмоль/кг) при низкой осмоляльности мочи — 302 мОсмоль/кг (300–1200 мОсмоль/кг), что подтвердило наличие ЦНД, терапия десмопрессином была возобновлена.

Вторичная надпочечниковая недостаточность (кортизол утром — 663 нмоль/л (171–536), АКТГ утром — 78 пг/мл) и вторичный гипотиреоз (свТ4 — 12,3 пмоль/л (9–19), ТТГ — 0,83 мМЕ/л (0,25–3,5) были исключены.

По данным МРТ головного мозга с контрастным усилением: МР-картина частично «пустого» турецкого седла, отсутствие сигнала от нейрогипофиза. Таким образом, подтвержден центральный генез сохраняющегося несахарного диабета (рис. 5, 6).

**Figure:**
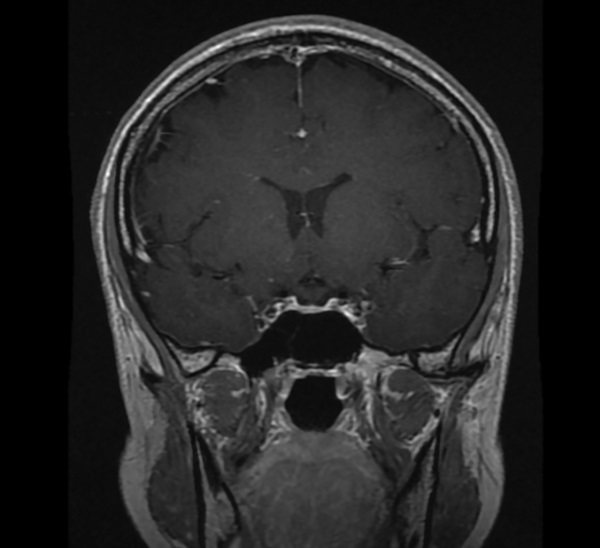
Рисунок 5. МРТ головы с контрастным усилением, Т1-взвешенное изображение, коронарная (фронтальная) проекция. МР-картина частично «пустого» турецкого седла.

**Figure:**
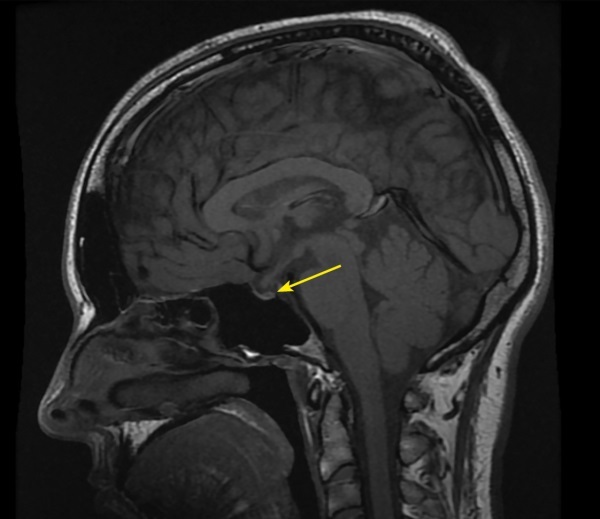
Рисунок 6. МРТ головы с контрастным усилением, Т1-взвешенное изображение,сагиттальная проекция. МР-картина частично «пустого» турецкого седла. Отсутствие сигнала от нейрогипофиза (изменения указаны стрелкой).

Кроме того, дополнительное обследование пациентки через 6 лет после родов позволило своевременно выявить злокачественное новообразование щитовидной железы (папиллярный рак, классический подтип, pT1bN0M0) и провести хирургическое лечение в объеме правосторонней гемитиреоидэктомии.

Таким образом, по результатам обследования установлен диагноз:

«Центральный несахарный диабет в исходе гипофизита, ассоциированного с беременностью от 2017 г., медикаментозная компенсация. Состояние после правосторонней гемитиреоидэктомии по поводу аденокарциномы правой доли щитовидной железы pT1bN0M0. Левосторонний узловой зоб 0 степени (ВОЗ)».

В связи с удовлетворительным самочувствием пациентки на фоне приема десмопрессина 15 мкг 2 раза в сутки рекомендовано продолжить терапию в прежней дозе с соблюдением адекватного питьевого режима. Пациентка выписана под динамическое наблюдение эндокринологом.

## ОБСУЖДЕНИЕ

В 1942 г. H. Blotner и P. Kunkel описали некоторые из самых ранних случаев несахарного диабета во время беременности, включая сообщения, предположительно относящиеся к 1790-м годам [11]. Десятилетия наблюдений показали, что существует множество причин развития НД во время беременности. Как было указано ранее, НД во время гестации часто развивается в результате повышения активности вазопрессиназы, секретируемой плацентой, и в таком случае носит название гестационного несахарного диабета, но также может быть следствием различных патологических процессов в головном мозге (центральный несахарный диабет) или резистентности почек к действию вазопрессина (нефрогенный несахарный диабет) [2].

Гестационный НД беременных (ГНД), также называемый транзиторным НД, является наиболее распространенным типом НД в этой группе пациенток [12]. Обычно при ГНД характерные симптомы (полиурия, полидипсия) отмечаются уже в первом триместре, но чаще достигают пика в конце второго или третьего триместра беременности по мере созревания и увеличения массы плаценты. Патогенез ГНД обусловлен повышением уровня плацентарной вазопрессиназы, что приводит к снижению циркулирующего вазопрессина (антидиуретический гормон, АДГ) на 80–85% [13], развитию НД с характерным разрешением всех симптомов вскоре после родоразрешения [14]. Помимо повышенной активности вазопрессиназы, физиологическая гипертрофия и гиперплазия передней доли гипофиза при беременности могут приводить к компрессии задней доли гипофиза и снижению уровня АДГ. Кроме того, при поражении печени, в ряде случаев осложняющем течение беременности (например, острая жировая дистрофия печени, HELLP-синдром), нарушение печеночной дезактивации вазопрессиназы также приводит к снижению уровня АДГ [15].

Тем не менее стоит отметить, что ГНД встречается достаточно редко и требует проведения дифференциальной диагностики с другими причинами НД у беременных [16].

Так, центральный НД (ЦНД) при беременности развивается в результате недостаточного синтеза и/или высвобождения АДГ и может манифестировать во время беременности за счет повышенной потребности в АДГ. В то время как ГНД обычно клинически проявляется только во 2-м и 3-м триместрах, когда уровни плацентарной вазопрессиназы достигают пика, ЦНД следует заподозрить, когда признаки и симптомы НД появляются на более ранних сроках беременности [1]. Причины ЦНД во время беременности могут быть разными, от генетических синдромов (например, синдром Вольфрама) до приобретенных форм (черепно-мозговая травма, хирургическое вмешательство, аутоиммунные (лимфоцитарный гипофизит), инфильтративные, опухолевые и инфекционные процессы в гипофизе и гипоталамусе) [17]. В редких случаях выявляется и послеродовый НД в рамках синдрома Шиена, связанного с ишемическими процессами в гиперплазированном гипофизе на фоне кровопотери в родах [18].

Нефрогенный НД (ННД) при беременности обусловлен нечувствительностью почек к действию АДГ и так же, как ЦНД, может манифестировать во время беременности за счет повышенной потребности в АДГ. В основе ННД могут лежать генетические и приобретенные причины. Так, например, Х-сцепленная рецессивная мутация гена AVPR2 на хромосоме Xq28 составляет 90% наследственного нефрогенного несахарного диабета [19]. Клиническая картина у женщин может варьировать от бессимптомного носительства мутации до субклинического НД. Другие наследственные и приобретенные заболевания почек также могут привести к манифестации нефрогенного НД во время беременности [1][20].

Подходы к лечению гестационного и центрального несахарного диабета у беременных существенно не различаются и предполагают терапию препаратами десмопрессина (устойчивого к действию вазопрессиназы в отличие от эндогенного вазопрессина). В то же время терапия нефрогенного НД у беременных обычно заключается в поиске и устранении причины НД [1].

В контексте вышеописанного случая с учетом достаточно характерной клинической картины и данных анамнеза, ответа на лечение глюкокортикоидами и десмопрессином, а также специфических изменений на МР-изображениях, вероятнее всего, имел место ЦНД в исходе аутоиммунного гипофизита.

Гипофизит — это редкое заболевание гипофиза, характеризующееся неопухолевой инфильтрацией его ткани, увеличением объема, приводящее к нарушению его функций. Распространенность гипофизита оценивается как 1 новый случай на 7–9 млн населения в год [21]. Выделяют первичную (вследствие аутоиммунного воспаления гипофиза) и вторичную (вследствие системных заболеваний или применения лекарственных средств) формы гипофизита [22].

Аутоиммунный гипофизит, как одна из форм заболевания, характеризуется инфильтрацией ткани гипофиза лимфоцитами, плазматическими клетками, эозинофилами, макрофагами и нейтрофилами, что приводит к фиброзной дистрофии железистой паренхимы и сопровождается гипофизарной дисфункцией различной степени [23].

Развитие аутоиммунного гипофизита во многих случаях ассоциировано с беременностью и родами, причем особенно характерно появление клинических симптомов во 2-м или 3-м триместре беременности или в первые 2 месяца после родов, однако потенциально лимфоцитарный гипофизит может манифестировать на любом сроке беременности [3].

Наиболее распространенной формой аутоиммунного гипофизита является лимфоцитарный гипофизит [24]. Долгое время термины аутоиммунный и лимфоцитарный гипофизит считались синонимами [25]. Однако с увеличением информации о гистологических различиях при гипофизите выяснилось, что лимфоцитарный гипофизит на самом деле представляет собой лишь одну из разновидностей аутоиммунного. К другим вариантам относятся гранулематозный, ксантоматозный, некротический, IgG4-опосредованный и медикаментозный гипофизит [24]. Поскольку подавляющее большинство случаев аутоиммунного гипофизита составляет именно лимфоцитарный, довольно часто в литературе взаимозаменяемо упоминаются эти два термина [25].

Гипофизит может поражать переднюю долю (аденогипофизит, 65% всех случаев) с выпадением ее тропных функций, заднюю долю и ножку гипофиза (инфундибулонейрогипофизит, 10% случаев) или вовлекать в патологический процесс весь гипофиз (пангипофизит, 25% случаев). В связи с этим клиническая картина лимфоцитарного гипофизита весьма вариабельна [3]. E. Thodou и соавт. в 1995 г. описали 16 пациентов с лимфоцитарным гипофизитом, причем в 63% случаев отмечали дисфункцию передней доли гипофиза, у 56% наблюдаемых — нарушение полей зрения и головные боли (как следствие масс-эффекта), у 38% — гиперпролактинемию, а у 19% пациентов — развитие НД [26]. По данным другого исследования, проведенного с участием 492 пациентов, различные проявления масс-эффекта (головные боли или нарушения зрения, парезы III, IV или VI пар черепных нервов) встречались наиболее часто (58%), также отмечались симптомы гипопитуитаризма (44%), при этом НД выявлялся в 31% случаев, а гиперпролактинемия — в 18% [27].

В рамках гипопитуитаризма при лимфоцитарном аденогипофизите, согласно данным литературы, наиболее часто выявляется дефицит АКТГ, за которым следуют выпадение секреции ТТГ, гонадотропных гормонов и пролактина [3].

Как было сказано ранее, лимфоцитарный инфундибулонейрогипофизит затрагивает преимущественно заднюю долю и ножку гипофиза, сопровождается клинической картиной НД и встречается значительно реже, чем аденогипофизит [2].

Вероятно, именно эта форма гипофизита наблюдалась у описанной нами пациентки, однако с учетом однократно полученных данных анализов с низким уровнем базального кортизола в крови утром, низконормальным уровнем свТ4 при сниженном уровне ТТГ нельзя исключить вовлечение в патологический процесс (вероятно, в меньшей степени) и аденогипофиза, которое, тем не менее, носило транзиторный характер. Стоит упомянуть о некоторых ограничениях при интерпретации гормональных исследований в данном случае. Снижение уровня ТТГ могло быть следствием перенесенного в первом триместре транзиторного тиреотоксикоза (данные об уровне ТТГ в первом триместре пациентка не предоставила). Однако сохраняющееся к 18–19 неделе беременности снижение уровня ТТГ, особенно с учетом низконормального уровня св.Т4, все же заставляет в первую очередь думать о развившемся вторичном гипотиреозе. Несмотря на то, что для беременных пациенток не разработаны референсные интервалы по базальному уровню кортизола, его существенно сниженный уровень (39,0 нмоль/л) также свидетельствует о вероятном транзиторном вторичном гипокортицизме.

Чаще всего инфундибулонейрогипофизит прогрессирует от воспаления до фиброза и последующей атрофии ткани нейрогипофиза, что в итоге проявляется в виде синдрома «пустого» турецкого седла со стойким гипопитуитаризмом/НД [28].

Характерная МР-картина при гипофизите — симметричное увеличение объема гипофиза (за счет увеличения его размеров), диффузная неоднородность сигнала от ткани аденогипофиза, кистозные изменения структуры передней доли гипофиза разной степени выраженности, а также активное накопление контрастного препарата прилежащей твердой мозговой оболочкой с формированием «дурального хвоста». В ряде случаев могут наблюдаться изменения структуры хиазмы и зрительных трактов (гиперинтенсивный МР-сигнал на Т2-взвешенных изображениях) [22].

При вовлечении в патологический процесс задней доли гипофиза обычно выявляется отек ее ткани, утолщение ножки гипофиза >3 мм на уровне срединного возвышения гипоталамуса, потеря характерного гиперинтенсивного сигнала от задней доли гипофиза [29]. Известно, что при гипофизите МР-картина часто расценивается как объемное образование гипофиза. A. Gutenberg и соавт. разработали рентгенологическую шкалу, позволяющую отличить аутоиммунный гипофизит от аденом гипофиза, и выделили 8 значимых предикторов, которые позволили бы правильно различать два этих состояния [29]. Появление симптомов на поздних сроках беременности, увеличенный и гомогенный в доконтрастную фазу гипофиз, потеря сигнала от задней доли гипофиза, утолщение ножки гипофиза свидетельствуют в пользу гипофизита [25][30], однако не всегда эти признаки являются специфичными, и некоторые из них также могут встречаться при объемных образованиях и инфильтративных процессах хиазмально-селлярной области, что создает определенные диагностические сложности [3]. В представленном нами случае на МР-изображениях первоначально описывалось объемное образование хиазмально-селлярной области, что, как указано выше, может наблюдаться и при гипофизите, но в то же время отмечались характерные для гипофизита признаки (утолщение ножки гипофиза, сохраняющееся отсутствие сигнала от нейрогипофиза).

Биопсия гипофиза является наиболее надежным способом диагностики лимфоцитарного гипофизита, однако этот метод является инвазивным и проводится только в отдельных случаях, когда диагноз сомнителен, и результаты биопсии могут повлиять на тактику лечения [2]. При отсутствии показаний к хирургическому лечению диагностика гипофизита основывается на клинических, лабораторных и рентгенологических данных [22].

Предпринимаются множественные попытки идентифицировать и использовать в практике специфические для аутоиммунного гипофизита антитела в качестве дополнительного диагностического маркера заболевания [3]. Так, в 2015 г. S. Iwama и соавт. [31] и K. Sakurai и соавт. [32] попытались использовать антитела к рабфилину-3А в качестве маркера лимфоцитарного инфундибулонейрогипофизита с развитием НД в третьем триместре беременности. Однако исследование титров антител к рабфилину-3А и других антигипофизарных антител в настоящее время в клинической практике все еще ограничено.

Большинство случаев лимфоцитарного гипофизита являются саморазрешающимися со спонтанным исчезновением офтальмологических и неврологических симптомов, связанных с компрессией структур турецкого седла. В то же время многим пациентам требуется долгосрочная заместительная терапия гипопитуитаризма [33]. Лечение гипофизита преимущественно направлено на замещение утраченных функций гипофиза и/или купирование симптомов масс-эффекта (головная боль, зрительные нарушения, парезы черепных нервов) [25]. Пульс-терапия глюкокортикоидами наиболее часто применяется при гипофизитах с выраженными головными болями, зрительными нарушениями и гипопитуитаризмом, приводя к восстановлению функции передней и задней долей гипофиза, уменьшению отека селлярной области и ножки гипофиза [34]. Другие иммуносупрессоры (ритуксимаб, азатиоприн, метотрексат и циклоспорин А) также в ряде случаев демонстрировали эффективность при гипофизите [24][35]. Хирургическое лечение обычно рассматривается в тяжелых или опасных для жизни случаях с выраженным нарушением полей зрения, парезом черепных нервов или при отсутствии ответа на медикаментозное лечение [36]. На фоне консервативного лечения восстановление функции гипофиза происходит в 27% случаев, а рентгенологическая регрессия отмечается в 46% [3].

Таким образом, в контексте описанного нами случая характерная клиническая картина с офтальмологическими нарушениями, выраженными головными болями, купировавшимися на фоне терапии дексаметазоном, и, конечно, развитие изолированного стойкого НД в совокупности со специфическими МР-изменениями с потерей сигнала от нейрогипофиза позволяют предположить перенесенный во время беременности инфундибулонейрогипофизит.

## ЗАКЛЮЧЕНИЕ

Вышеописанный клинический случай демонстрирует редкий вариант развития гипофизита во время беременности с изолированным стойким нарушением функции задней доли гипофиза и развитием ЦНД. Следует помнить о гипофизите как одной из возможных причин гипопитуитаризма у беременных пациенток и во всех случаях исключать дефицит тропных гормонов гипофиза, так как невыявленные вторичная надпочечниковая недостаточность и вторичный гипотиреоз могут угрожать жизни матери и ребенка во время или после родов. Знание особенностей МР-диагностики и подходов к лечению при гипофизите позволяет избежать ненужного в большинстве случаев оперативного лечения по поводу образований гипофиза, что особенно важно при ведении беременных пациенток. Определенные диагностические трудности и небольшая распространенность гипофизита среди беременных требуют высокой осведомленности среди врачей-эндокринологов и акушеров-гинекологов относительно течения, диагностики и лечения данного заболевания.

## ДОПОЛНИТЕЛЬНАЯ ИНФОРМАЦИЯ

Источники финансирования. Обследование и лечение пациентки осуществлялось за счет средств, выделенных по Гранту №121030100034-1 «Эндокринно-опосредованные нарушения осмотического гомеостаза: изучение этиологических и патогенетических факторов, разработка персонализированных подходов дифференциальной диагностики».

Конфликт интересов. Авторы декларируют отсутствие явных и потенциальных конфликтов интересов, связанных с содержанием настоящей статьи.

Участие авторов. Все авторы одобрили финальную версию статьи перед публикацией, выразили согласие нести ответственность за все аспекты работы, подразумевающую надлежащее изучение и решение вопросов, связанных с точностью или добросовестностью любой части работы.

Согласие пациента. Пациентка добровольно подписала информированное согласие на публикацию персональной медицинской информации в обезличенной форме.
